# Complete lung agenesis caused by complex genomic rearrangements with neo-TAD formation at the SHH locus

**DOI:** 10.1007/s00439-021-02344-6

**Published:** 2021-08-26

**Authors:** Uirá Souto Melo, Juliette Piard, Björn Fischer-Zirnsak, Marius-Konstantin Klever, Robert Schöpflin, Martin Atta Mensah, Manuel Holtgrewe, Francine Arbez-Gindre, Alain Martin, Virginie Guigue, Dominique Gaillard, Emilie Landais, Virginie Roze, Valerie Kremer, Rajeev Ramanah, Christelle Cabrol, Frederike L. Harms, Uwe Kornak, Malte Spielmann, Stefan Mundlos, Lionel Van Maldergem

**Affiliations:** 1 Max Planck Institute for Molecular Genetics, RG Development and Disease, Berlin, Germany; 2 Institute of Medical Genetics and Human Genetics, Charité-Universitätsmedizin Berlin, Corporate Member of Freie Universität Berlin and Humboldt-Universität zu Berlin, Berlin, Germany; 3 Centre de Génétique Humaine, Université de Franche-Comté, Besançon, France; 4 Berlin Institute of Health at Charité-Universitätsmedizin Berlin, Berlin, Germany; 5 Departments of Obstetrics and Gynecology, Pathology, and Biology, University Hospital, University of Franche-Comte, Besançon, France; 6 Department of Obstetrics and Gynecology, University Hospital Grenoble-Alpes, Grenoble, France; 7 Department of Medical Genetics, University Hospital, University Champagne-Ardennes, Reims, France; 8 Laboratory of Cytogenetics, University Hospital, University of Strasbourg, Strasbourg, France; 9 Institute of Human Genetics, University Medical Center Hamburg-Eppendorf, Hamburg, Germany; 10 Institute of Human Genetics, Universitätsmedizin Göttingen, Göttingen, Germany; 11 Institute of Human Genetics, University of Lübeck, Lübeck, Germany; 12 Center of Clinical Investigation (CIC), National Institute of Health and Medical Research (INSERM), CHU, Besançon, France; 13 Present Address: Department of Medical Genetics, University Hospital, Geneva, Switzerland

## Abstract

During human organogenesis, lung development is a timely and tightly regulated developmental process under the control of a large number of signaling molecules. Understanding how genetic variants can disturb normal lung development causing different lung malformations is a major goal for dissecting molecular mechanisms during embryogenesis. Here, through exome sequencing (ES), array CGH, genome sequencing (GS) and Hi-C, we aimed at elucidating the molecular basis of bilateral isolated lung agenesis in three fetuses born to a non-consanguineous family. We detected a complex genomic rearrangement containing duplicated, triplicated and deleted fragments involving the SHH locus in fetuses presenting complete agenesis of both lungs and near-complete agenesis of the trachea, diagnosed by ultrasound screening and confirmed at autopsy following termination. The rearrangement did not include *SHH* itself, but several regulatory elements for lung development, such as MACS1, a major *SHH* lung enhancer, and the neighboring genes *MNX1* and *NOM1*. The rearrangement incorporated parts of two topologically associating domains (TADs) including their boundaries. Hi-C of cells from one of the affected fetuses showed the formation of two novel TADs each containing *SHH* enhancers and the *MNX1* and *NOM1* genes. Hi-C together with GS indicate that the new 3D conformation is likely causative for this condition by an inappropriate activation of *MNX1* included in the neo-TADs by MACS1 enhancer, further highlighting the importance of the 3D chromatin conformation in human disease.

**Supplementary Information:**

The online version contains supplementary material available at 10.1007/s00439-021-02344-6.

## Introduction

Complete absence of one or both lungs is an extremely rare malformation with an estimated birth prevalence below 1/10^7^ if we estimate that only a dozen of reports have been published so far, mostly occurring unilaterally (Ostör et al. 1978; Mardini et al. 1985; Spear et al. 1987; Engellenner et al. 1989; Podlech et al. 1995; Kayemba-Kay's et al. 2014). This extreme deleterious phenotype is caused by a failure in the proper formation of the lung buds derived from the foregut with no detectable respiratory tissue (lung agenesis) or by their deficient proliferation and branching leading to the development of short, blind ending bronchi (lung aplasia) during the early embryonic stage. Understanding how genetic variants can cause different lung malformations is a major goal for dissecting molecular mechanisms during embryogenesis.

During lung development, the conducting airways are formed first, followed by the formation of alveolae. The left and right lungs have their own anlage, derived from the anterior foregut endoderm, a tissue where also thyroid, esophagus, and liver are originated from (Kadzik and Morrisey 2012). In humans, in the late 4th week after conception, the embryonic stage of lung development starts with the formation of two outpouchings in the ventral wall of the foregut termed lung buds, that start to proliferate in repetitive circles of growth and branching (Schittny 2017). While these buds, that form the later respiratory epithelium, are derived from the endoderm, they grow and branch inside of mesoderm derived tissues. An intensive crosstalk between endodermal and mesenchymal mesodermal cells is required for the formation and growth of the lung buds. This crosstalk also regulates the simultaneous development of the mesoderm derived cardiopulmonary vasculature together with the respiratory epithelium (Schittny 2017; Kimura and Deutsch 2007; Swarr and Morrisey 2015).

Here we evaluate a family with three fetuses affected by complete lung agenesis suggestive of an autosomal recessive inheritance pattern. Combining array CGH with genome sequencing (GS) and chromosome conformation capture (Hi-C) data, we were able to dissect this case in its complexity. We identified a complex rearrangement at the SHH locus that is proposed to result in the extreme lung condition in our cases. To the best of our knowledge, no study has been done to interrogate the molecular cause of isolated lung agenesis in humans.

## Methods

### Subjects, collected samples and cell culture

**Fig. 1:**
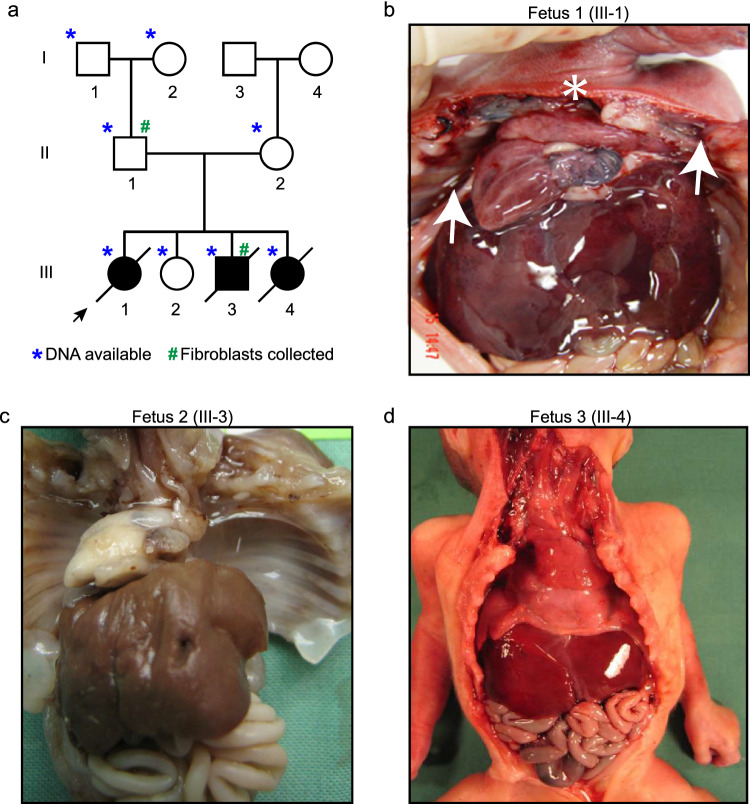
Family tree and phenotypes of the three affected fetuses. **a** Family pedigree. DNA samples were available from individuals marked with blue asterisk (*) and fibroblast from those marked with green number symbol (#). **b** Autopsy of Fetus 1 shows absence of the lungs. White arrows indicate a very small bud of lung. White asterisk indicates the location where trachea should have been observed. Other organs are present. **c** Autopsy of Fetus 2 showing lung agenesis. Gallbladder was absent. **d** Similar to Fetus 1, empty thorax is observed in Fetuses 2 and 3

Healthy parents provided written informed consent to all subjects enrolled in this study. All biopsies and molecular testing were performed after obtaining written consent of the patients in accordance with the rules of Helsinki. Blood samples were collected from grandparents (I-1 and I-2), healthy parents (II-1 and II-2) and one healthy sibling (III-2). We collected fetal material from three affected individuals (III-1, III-3 and III-4; Fig. 1a).

Fibroblast cell lines were established from skin biopsies of the father (II-1) and the Fetus 2 (III-3). Fibroblasts were cultured in DMEM (Thermo Fisher Scientific) supplemented with 10% fetal bovine serum (FBS; Thermo Fisher Scientific), 1% l-glutamine (Thermo Fisher Scientific) and 1% penicillin–streptomycin (Thermo Fisher Scientific). Fibroblasts from two unrelated healthy individuals were used as controls.

### DNA and RNA extraction, and quantitative PCR analyses (qPCR and RT-qPCR)

DNA and RNA were extracted using DNeasy Blood and Tissue Kit (Qiagen) and RNeasy Mini Kit (Qiagen), respectively. qPCR was performed to measure the copy number of fragments detected by array CGH. RT-qPCR was performed to measure the expression of candidate genes located at the SHH locus: *SHH*, *RNF32*, *LMBR1*, *NOM1*, *MNX1* and *UBE3C*. Primer sequences are available upon request. qPCR and RT-qPCR were performed using the PowerUp™ SYBR® Green Master Mix (Thermo Fisher) in the QuantStudio 6 Flex Real-Time PCR System, 384-well (Applied Biosystems). Copy number and gene expression were calculated using 2^−ΔΔCT^ method (Schmittgen and Livak 2008). Each experiment was performed once with three technical replicates per sample.

### Genomic screening

Microarray-based comparative genomic hybridization (array CGH) was performed in DNA from the healthy father (II-1; blood) and two fetuses (III-1 and III-3) using the 1 M arrays (Agilent, Santa Clara, CA). Copy number variation (CNV) detected by array CGH were further assessed by qPCR of the following fragments: A (15 Kb duplication, chr7:156,181,014–156,196,660 × 3); B (449 kb triplication, chr7:156,196,660–156,645,844 × 4); C (207 kb duplication, chr7:156,645,844–156,853,796 × 3), D (neutral copy, 9.8 kb, chr7:156,853,796–156,863,687) and E (61 kb deletion, chr7:156,863,687–156,924,827 × 1), also calibrator regions A’ and E’, located up- and downstream the rearrangement.

Targeted enrichment and massively parallel sequencing were performed on genomic DNA from the healthy father (blood) and two fetuses (III-1 and III-3). Enrichment of the Exome was performed according to the manufacturer’s protocols using the Nextera Enrichment Kit (62 Mb) (Illumina). Captured libraries were then loaded onto the 2500 platform (Illumina). The following genes referenced in OMIM as associated to agenesis, aplasia or hypoplasia of the lung in humans were analyzed for rare, likely pathogenic variants: *FOXF1* (#265380); *ZFPM2*, *TBX4* and *FGF10* (#265430).

Genome sequencing was performed in the healthy father (blood) and Fetus 1 (III-1) to validate the CNVs detected by array CGH and to identify the breakpoints at the base pair level to disentangle the nested complex rearrangement. Details of the protocol and pipeline are described previously (Melo et al. 2020).

### Hi-C analysis

Hi-C libraries were processed with our in-house pipeline based on the previously published in situ protocol (Rao et al. 2014). Briefly, ~ 1 million cells were fixed in 2% formaldehyde, lysed and digested overnight with DpnII enzyme (New England BioLabs). Next, digested DNA ends were marked with biotin-14-dATP (Thermo Fisher Scientific) and ligated overnight using T4 DNA ligase (New England BioLabs). DNA was sheared to fragments of 300–500 bp for library preparation and biotin-filled DNA fragments were pulled down using Dynabeads MyOne Streptavidin T1 beads (Thermo Fisher Scientific). The DNA was prepared for short reads sequencing by ligating adaptors to the DNA fragments, using the NEBNext Multiplex Oligos for Illumina kit (New England BioLabs). Libraries were deep sequenced (~ 240 Million fragments) in a 75 bp paired-end run on a HiSeq4000 (Illumina). Paired-end sequencing data were processed using the Juicer pipeline (Durand et al. 2016). A detailed protocol is described elsewhere (Melo et al. 2020).

## Results

### Clinical evaluation and autopsies of three fetuses revealed complete bilateral lung agenesis

The first pregnancy of a Caucasian woman aged 25 (II-2), unrelated to her 27-year-old partner (II-1), was remarkable for the detection of complete bilateral lung agenesis by ultrasound during the 2nd trimester of pregnancy (Fig. 1a and b). The next three pregnancies disclosed one healthy girl (III-2) and two other fetuses, a male and a female, also presenting the same severe lung phenotype.

The first fetus, a female (III-1), examined after termination of the pregnancy at 22 weeks indicated growth parameters within the normal range (510 g). Autopsy of the thorax revealed complete absence of the lung tissue (Fig. 1b). The heart was medially displaced and did not present any malformation. A complete atresia of a shortened trachea was observed, its distal part being measured at 5 mm of laryngeal structures. Left and right pulmonary artery were absent. There was no other malformation of the viscera, face or limbs. Histology of thymus and esophagus were normal.

The second fetus, a male (III-3), was examined after termination of the pregnancy at 24 weeks. His weight was 710 g (mean). Autopsy of the thorax revealed neither lung nor trachea in this fetus (Fig. 1c). Crown-rump distance was 22.5 cm (NR). 2 mm under the larynx, a small dimple in a structure that resembles a tracheal bud is seen without any lumen. Similar to Fetus 1 (III-1), integrity of the trunk of the pulmonary artery was observed while its branches were absent. There was no pulmonary vein return in the left atrium. Penis length was at lower limit of normal range (9 mm) and the gallbladder was absent. Histology was unremarkable.

The third fetus, a female (III-4) was examined after termination at 17 weeks of gestation. This fetus had normal growth parameters and similar autopsy findings with the other two fetuses (Fig. 1c).

Complete lung agenesis is an ultra-rare event and led us to propose a genetic cause of recurrence in the three evaluated conceptuses.

### Genetic screening revealed a complex rearrangement on 7q36.3 in all three fetuses

Chromosomal anomalies were ruled out by performing standard karyotyping in both parents and in the three fetuses (data not shown). Based on the pedigree (Fig. 1a), an autosomal recessive mode of inheritance was first taken into consideration. We performed exome sequencing (ES) in Fetuses 1 and 2 and searched for mono- or biallelic rare variants in known genes related to agenesis, aplasia or hypoplasia of the lung in humans (see "Methods") and in the orthologue genes in mouse (*ALDH1A2*, *FGFR2*, *TCF21*, *BCLAF1*, *MEK1* and *MEK2*) (Park et al. 2019; Arman et al. 1999; McPherson et al. 2009; Wang et al. 2006; Boucherat et al. 2014). ES failed to identify potential deleterious variants in homozygosis or compound heterozygosis in these genes. Furthermore, no other potential candidates neither recessive, nor dominant de novo were identified.

**Fig. 2:**
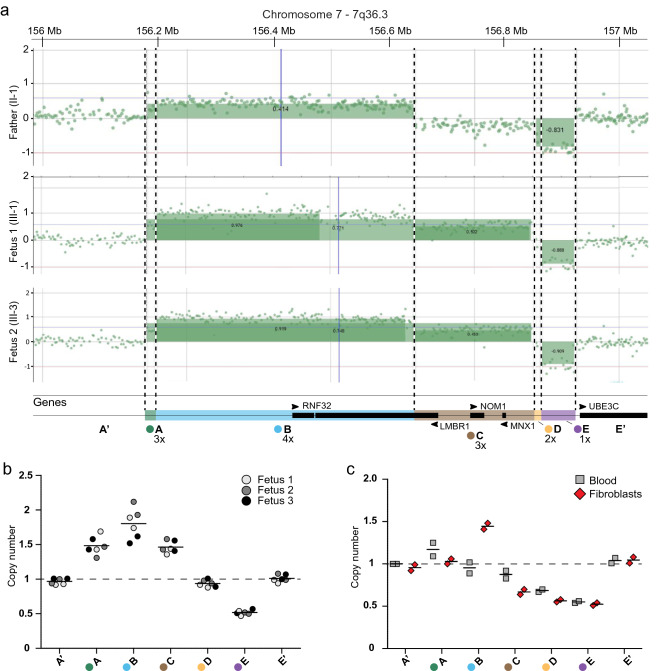
Copy number variation at the 7q36.3 locus. **a** Array CGH detected a complex rearrangement involving duplication, triplication and deletion in two fetuses. This variant is also presented in mosaic state in the father. The fragments A-E have different copy number values: A (3×; green), B (4×; blue), C (3×; brown), D (2×; orange) and E (1×; purple). **b** qPCR in fibroblasts confirmed the copy number values in all three fetuses. A′ and E′ fragments are copy number neutral up- and downstream of the complex rearrangement and were used as control. **c** qPCR in blood (grey squares) and fibroblasts (red 45° squares) samples from the healthy father showed different copy numbers for the A–D fragments, suggesting mosaicism. The E fragment is deleted in heterozygosis in both father’s blood and fibroblasts samples

Next, we performed array CGH in blood samples from the healthy father and in fetal DNA from Fetuses 1 and 2. Although no homozygous CNVs were identified in these samples, we detected a complex genomic rearrangement (CGR) on 7q36.3 shared by both fetuses, containing duplicated, triplicated and deleted fragments (Fig. 2a). Since this structural variant (SV) is located ~ 580 kb downstream of *SHH*, we suspected a long-range effect on this gene implicated in lung bud development being causative. *Shh* is involved in lung development and a knockout mouse model for *Shh* exhibits lung malformations (Chiang et al. 1996; Litingtung et al. 1998). This variant was also detected in the genitor, but it showed a distinct pattern in the array CGH in comparison to the fetuses (Fig. 2a). Given a less than 1.5 × copy number gain for the region containing the *RNF32* gene, we assume a mosaic status of the duplication in the father.

For clarity, we named the up-(A′) and the downstream (E′) copy neutral regions surrounding the SV, as well as five fragments (A–E) defined according to their copy number value. The B and C fragments contain the *RNF32*, *LMBR1*, *NOM1* and *MNX1* genes, while the remaining fragments do not contain known protein coding genes (Fig. 2a). qPCR confirmed the findings from array CGH. Thus, the CGR consists of a duplication of the A and C fragments (~ 1.5×), a triplication of B (~ 1.75×) and a deletion of E (~ 0.5×) in all three of the fetuses’ samples (Fig. 2b). qPCR performed in blood samples from the father detected ~ 1.2 copies of A and no extra copy of B (Fig. 2c) and the C and D fragments were partially reduced (~ 0.85 and ~ 0.7×, respectively), suggesting that not only just a fraction of cells harbors the complex rearrangement, but also a third allele without (C), (D) and (E) fragments is very likely to be present in the father’s samples (Fig. S1c). DNA from the father’s fibroblasts showed a duplication of B (~ 1.5×) and a reduction of C (~ 0.67×) and D (~ 0.56×) (Fig. 2c), thus corroborating a mosaic status. The E fragment is deleted in heterozygous state (~ 0.5×) in both, the father’s blood and the fibroblasts samples. The remaining healthy relatives do not carry this variant (Fig. S1a). These results suggest that the father is likely mosaic for the A-D fragments, resulting in a lower copy number state for theses fragments, providing a possible explanation for the absence of a clinical phenotype.

### Expression analysis in fibroblast from one affected case, the healthy father and controls

We investigated the impact of the SV on gene expression in the available fibroblasts samples. *MNX1* and *SHH* are not expressed in this cell line according to our in-house fibroblast expression database; indeed, RT-qPCR did not detect both genes transcripts in all tested samples (data not shown). RT-qPCR from Fetus 2 revealed upregulation of *RNF32* (~ 4×) and *LMBR1* (~ 1.5×) (Fig. S1b). In the healthy father, we observed reduced *LMBR1* and *NOM1* expression, which correlates with absence of these genes in one allele (Fig. 2c; Figs. S1b and c). The two candidate genes showing differential gene expression in fibroblasts were *RNF32*, encoding a ring finger protein involved in protein-DNA or protein–protein interactions and thought to play a role in spermatogenesis (van Baren et al. 2002); and *LMBR1,* a widely expressed gene with putative membrane receptor function. Our interpretation is that gene dosage of both genes is unlikely to be involved in lung agenesis. Therefore, we suspected that ectopic gene expression due to positional effect (e.g., enhancer adoption) could be the pathomechanism involved in this disease.

### Genome sequencing alone is not able to fully reconstruct the der(7q) linear sequence

**Fig. 3:**
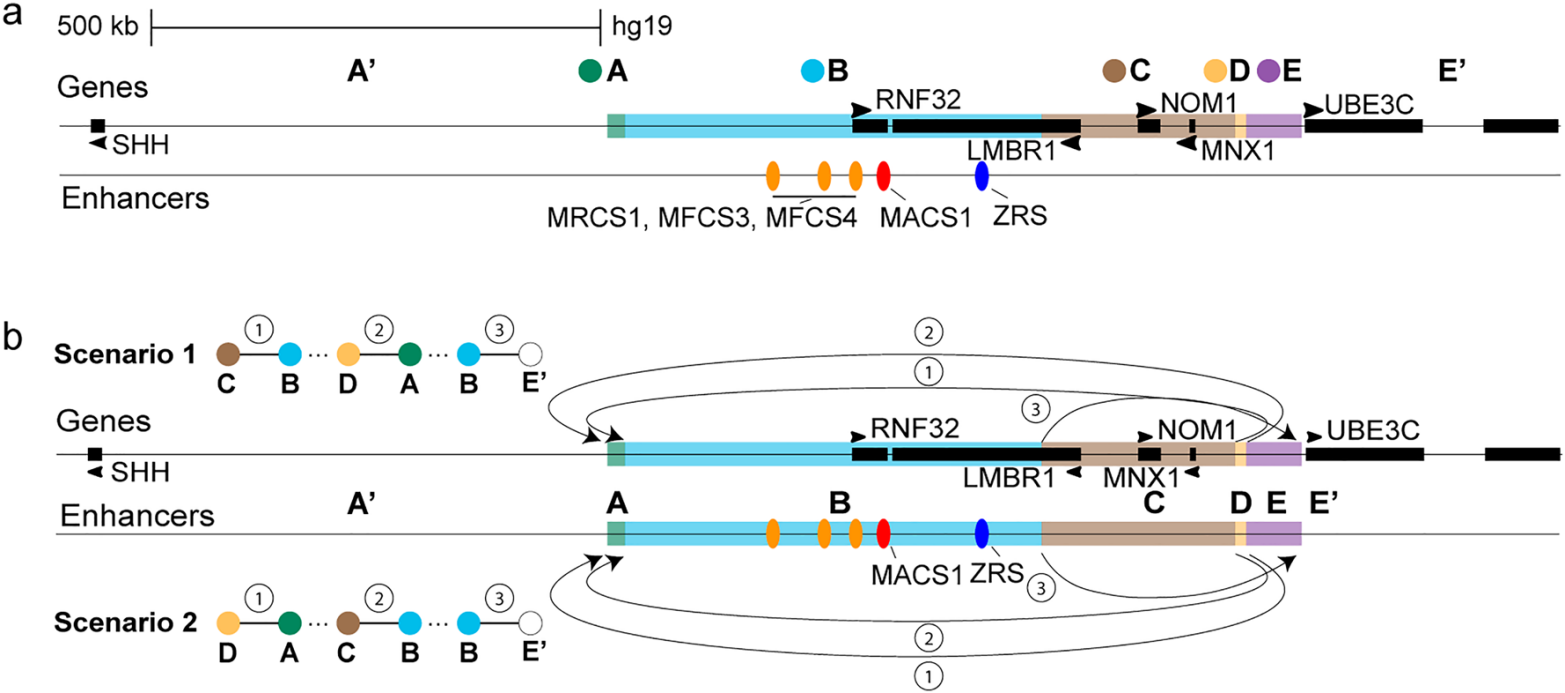
Genes and enhancers at the 7q36.3 region. **a** The A and D fragments have 15 and 9.8 kb length, respectively, and contain neither genes nor enhancers; the same is observed for the E fragment. The B and C fragments, sized 449 and 207 kb, respectively, contain several enhancers and two of them, MACS1 (red ellipse) and ZRS (blue ellipse), regulate *SHH* in a spatio-temporal manner. The other known enhancers are represented by orange ellipses. **b** Genome sequencing breakpoint analysis disclosed two possible linear sequence scenarios. Three interactions are observed by manual inspection of split-reads: D–A (1), C–B (2) and B–E′ (3)

We performed genome sequencing (GS) in samples from the healthy father (blood) and Fetus 2 to identify the breakpoints at the base pair level and solve the nested structure of the SV. We visually inspected the data for split-reads/chimeric read-pairs on 7q36.3 in both samples and observed breakpoints spanning reads for the D–A, C–B and B–E′ fragments (Figs. 3a and b). Note that few split-reads support the presence of the A–D fragments in the father, thus once again supporting mosaicism (Fig. S3). Based on GS data, the rearranged linear sequence could be reconstructed according to two hypothetical scenarios (i.e., Scenario 1 and 2; Fig. 3b and Fig. S2). Thus, the GS split-read analysis alone is not able to fully resolve the correct linear sequence of this CGR.

### Combined Hi-C and genome sequencing helps to resolve and interpret the complex rearrangement

**Fig. 4:**
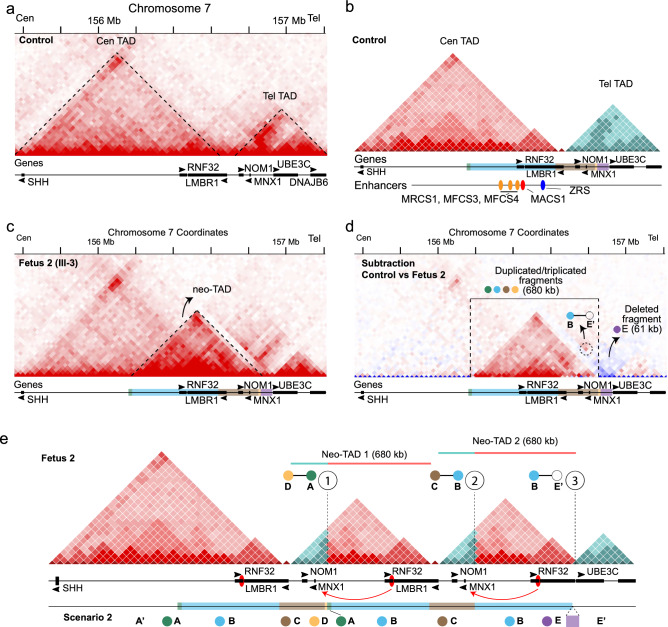
3D chromatin landscape at the SHH locus in healthy and affected fibroblasts. **a** Hi-C map of control fibroblast sample (25 kb resolution; raw data) showing the 3D landscape of the 7q36.3 locus. Genes (black rectangles; arrows indicate the orientation of the transcript) are listed below. **b** Schematic representation of TAD structures on the 7q36.3 region in a wild-type sample (the centromeric TAD in red and the telomeric one in blue). MACS1 and ZRS enhancers (colored in red and blue, respectively) are shown on the track below. Additional known oral and pharyngeal epithelium enhancers are shown in orange. **c** Hi-C map from fibroblasts the Fetus 2 revealed ectopic signal due to novel chromatin contacts. **d** Subtracted map shows the gain of new chromatin interaction in the fetus. Red: gain of contact, blue: loss of contact. **e** Schematic representation of the derivative 7q36.3 3D structure caused by the complex genomic rearrangement. Observe the formation of two neo-TADs, both allowing ectopic interaction of MACS1 with *MNX1* and *NOM1* promoters (red arrows). Based on our analysis, both scenarios 1 and 2 are compatible to be true, i.e., they would give roughly similar outcomes related to 3D genome architecture reconstruction; therefore both are likely for being causative. In this work, we choose Scenario 2 for merely illustration purpose

To understand the 3D genome landscape at the SHH locus, we analyzed fibroblasts Hi-C maps from an unrelated healthy control (Melo et al. 2020) and we observed two topologically associating domains (TADs) at this locus: (i) the SHH-TAD which contains the *SHH*, *RNF32* and *LMBR1* genes, (ii) a telomeric TAD comprising *NOM1*, *MNX1*, *UBE3C*, and *DNAJB6* (Fig. 4a and b). Importantly, sonic hedgehog (*Shh*) is a major developmental gene that controls cell survival and fate, and axial patterning in the vertebrate body plan. As shown in mouse mutants, *Shh* is required for the growth and differentiation of the esophagus, trachea and lung (Litingtung et al. 1998). The SHH chromatin domain (cen-TAD) contains several known cis-regulatory elements (CREs) that control diverse expression patterns, including MRCS1, MFCS4, and MACS1, which drive *Shh* expression in the epithelia of the larynx, lung and intestinal and urogenital tracts (Fig. S4a; Sagai et al. 2009). MACS1, MRCS1 and MFCS4 as well as the limb enhancer ZRS are located in the SHH-TAD and are physically insulated from the telomeric TAD by a boundary element.

Next, we performed Hi-C in fibroblasts of Fetus 2 (III-3) and the father (II-1) to solve the nested structure of the SV and simultaneously interpret the 3D genome architecture of this region. Hi-C revealed a similar ectopic chromatin interaction pattern in the father and the fetus (Fig. 4c and d; Figs. S4b and S4c), however, the pattern signal was weaker in the father than in the fetus, indicating a lower copy number of the rearranged fragments, in accordance with his mosaicism detected previously. Noteworthy, a wild-type copy of MACS1 is still located within the SHH-TAD, thus *SHH* seems to be unaffected by position effect of this SV.

The A and D fragments have 15 and 9.8 kb in length, respectively, and do not contain known enhancers. Also the E fragment (61 kb heterozygous deletion) is devoid of known cis-regulatory elements (Fig. 4b). The B and C fragments, sized 449 and 207 kb, respectively, contain several enhancers and two of them, MACS1 and ZRS, regulate *SHH* (Fig. 4b; Anderson et al. 2014). Based on these findings, we then hypothesized that a novel enhancer-promoter interaction caused by the repositioning of the lung enhancers into another TAD might lead to gene misexpression during primary lung buds development, likely causing the disease.

To better visualize the impact of the SV in the Hi-C map, we subtracted the Hi-C signal of the control and we observed a strong chromatin interaction of about 680 kb in the fetus (Fig. 4d). After subtraction, the direct contact of Fragments B–E′ and the 61 kb deletion (Fragment E) became visible. The simplest way of visualizing the derivative locus is drawing a schematic representation of the derivative landscape (Melo et al. 2020). Combining the linear sequence obtained from the GS data and adding on top the Hi-C information, we observed the formation of two new domains (neo-TADs) each about ~ 670–680 kb in size. In both neo-TADs, the MACS1 enhancer can physically interact with *MNX1* and *NOM1* (Fig. 4e). Interestingly, *MNX1* is known to upregulate Wnt/ß-catenin signaling and its downstream targets c-Myc and CCND1 (Yang et al. 2019).

Taken together, these data suggest that the 7q36.3 SV here identified creates two neo-TADs, thus allowing ectopic MACS1 contact with *MNX1 *and *NOM1* promoters in lung tissue, with a likely blocking effect on the WNT signaling pathway, resulting in absent development of primary air cells and eventually leading to the bilateral lung agenesis observed in the three fetuses.

## Discussion

Complete absence of the lung is an ultra-rare malformation described in only few cases worldwide. Pulmonary hypoplasia is more common and mostly occurs in association with fetal hydrops, congenital diaphragmatic hernia, oligohydramnios, or skeletal dysplasia; a broader phenotypic spectrum can also be observed and includes patients harboring coding and non-coding pathogenic variants (Reviewed in Nogee and Ryan 2021). Rare familial occurrence has been described (Anderson et al. 2014). In the present study, the exceptional recurrence of complete bilateral lung agenesis in three fetuses suggested a genetic basis. After applying a combination of several genetic/genomic screening methodologies, we identified a CGR on 7q36.3 shared by all three affected fetuses, presenting duplicated, triplicated and deleted fragments. Previous studies have shown that accurate detection and interpretation of complex rearrangements should be done by integration of different genomic technologies (Carvalho and Lupski 2016); indeed, the complexity of this SV was only resolved by a combined strategy consisting of array CGH, GS, and Hi-C. Our study further supports the utility of Hi-C for the detection, characterization and interpretation of complex genomic rearrangements. Hi-C was instrumental in the precise localization of the individual CNVs, but was also helpful in interpreting the effects of the rearrangements.

The pedigree of the enrolled family in this work was suggestive of autosomal recessive inheritance for the lung phenotype, but ES in the affected fetuses failed to identify mono- or biallelic rare variants in known genes related to agenesis, aplasia or hypoplasia of the lung, or other potential candidate genes (Park et al. 2019; Arman et al. 1999; McPherson et al. 2009; Wang et al. 2006; Boucherat et al. 2014). The candidate CGR identified here was present in the unaffected father, first resulting in its interpretation as a variant of unknown significance. Incomplete penetrance or mosaicism was considered as another possibility. Based on several experiments including array CGH, qPCR, RT-qPCR, GS and Hi-C, we were able to conclude that the father is mosaic for this SV in agreement with the observed inheritance pattern. Thus, the careful dissection of this complex rearrangement resulted in an explanation completely different from our original hypothesis.

SVs can interfere with chromatin folding if they disrupt TADs. TADs have been identified as regions in the genome that show high interaction in the 3D space of the nucleus (Rao et al. 2014). They are separated from each other by regions of low interaction, so called boundaries. The interactions measured by chromosome conformation capture (3C) methods such as Hi-C reflect the physical proximity of enhancers with the target promoters. Since Hi-C is a quantitative measure of proximity, it can also be used to identify rearrangements (Melo et al. 2020). Here, Hi-C together with array CGH and GS indicated a triplication of a region containing part of the SHH-TAD and thus *SHH* enhancers (B fragment) and a duplication of the boundary between the SHH-TAD and its neighboring TAD that contains the *MNX1* and *NOM1* genes (C fragment). As previously shown by us (Franke et al. 2016; Spielmann et al. 2018), duplications can result in the formation of novel chromatin domains, so called neo-TADs, if a boundary is included in the duplication. Therefore, regulatory elements are connected in the neo-TAD with genes that were previously separated. In the current case, the inclusion of the boundary suggests the generation of two neo-TADs each containing *SHH* enhancers and the *MNX1* and *NOM1* genes. However, we cannot exclude the involvement of other yet so far unknown lung enhancers in the region.

Sonic hedgehog (SHH) and its downstream effector GLIs are major players in determination of the fate of pulmonary bud cells (Warburton et al. 2005; Fernandes-Silva et al. 2017). *Shh* is widely expressed in the foregut endoderm and is specifically upregulated in the distal epithelium of the lung where branching is occurring (Miller et al. 2004). In *Shh* null mutants, the lungs form a rudimentary sac due to a failure of branching and growth after formation of the primary lung buds (Litingtung et al. 1998; Pepicelli et al. 1998). Furthermore, the deletion of two transcription factors mediating the *Shh* pathway, *Gli2* and *Gli3*, resulted in an absent formation of lung, trachea and esophagus in mice (Motoyama et al. 1998), further supporting the importance of this pathway in lung development. It is thus to be expected that the SHH-TAD contains enhancers that drive *Shh* expression during lung morphogenesis, and a cluster of such enhancers has been identified of which MACS1 has been shown to be essential for *Shh* expression in the laryngeal epithelia and lung buds in mice (Sagai et al. 2009; Sagai et al. 2017). Accordingly, MACS1 knockouts show defects in the respiratory organogenesis (Sagai et al. 2017). A more common finding than lung aplasia are tracheal malformations, sometimes in combination with tracheoesophageal fistulas (Evans et al. 1999). These malformations are observed in the well-described VATER association. After experimental induction of tracheoesophageal malformations in an animal model a dysregulation of *SHH* was found (Ioannides et al. 2003). Interestingly, a deletion of 7q35q36.3 was found in a case with VATER symptoms (PMID 27436264). It would be further interesting to perform Hi-C in such cases to learn more about the impact of the rearrangements on the *SHH* locus. It is also known that *SHH* mutations are associated with other congenital malformations, e.g., Holoprosencephaly 3 (MIM 142945), Microphthalmia with coloboma 5 (MIM 611638), Single median maxillary central incisor (MIM 147250), and Schizencephaly (MIM 269160), however, as expected, the symptoms detected in these disorders were not present/evaluated in our fetuses due to the preservation of the wild-type SHH-TAD.

The other TAD involved in the duplications contains *MNX1*, also called HLXB9, encoding a homeobox transcription factor expressed in lymphocytes, colon, stomach, small Intestine, pancreas, and pituitary gland (Harrison et al. 1999). Heterozygous mutations in *MNX1* cause Currarino triad (MIM #176450; Lynch et al. 1995; Ross et al. 1998), a condition characterized by partial sacral agenesis, a presacral mass, and anorectal malformation. Here we hypothesize that ectopic activation of this gene by the MACS1 or other lung enhancers could have catastrophic effects in the fine-tuned network of signaling molecules necessary for proper lung development. *MNX1* is known to upregulate Wnt/ß-catenin signaling and its downstream genes c-Myc and CCND1. An alteration of this pathway could lead to a failure in formation or outgrowth of the primary lung buds (Yang et al. 2019). Additionally, the long non-coding RNA (lncRNA) *MNX1-AS1* activates MAPK signaling, another important pathway which may be involved in proper lung development (Liu et al. 2019), for the reason that *Mek1*/*Mek2* double knockout presents pulmonary hypoplasia and other related phenotypes (Boucherat et al. 2014). A similar mechanism has been described for Acropectorovertebral syndrome (OMIM: #102510) in which misexpression of *PAX3* under the control of *EPHA4* enhancers causes a complex limb malformation (Lupiáñez et al. 2015). However, additional studies need to be performed to support the hypothesis of *MNX1* activation by MACS1 in lung tissue as the cause of lung agenesis in our patients.

In conclusion, the current observation provides the first example of congenital absence of lungs likely due to a TAD disorganization by a copy number variation. It further substantiates the importance of proper interpretation of CNVs above their positional constrains to elucidate the missing heritability. Thus, ectopic enhancer-promoter interaction could lead to *MNX1* activation in lung cells by MACS1, causing the lung agenesis in the three fetuses.

## Supplementary Information

Below is the link to the electronic supplementary material.

Supplementary file1 (PDF 630 KB)

## Data Availability

Data and materials are available upon request.
