# Supplementary material for: Complete lung agenesis caused by complex genomic rearrangements with neo-TAD formation at the SHH locus
Source: Hum Genet. 2021 Aug 26;140(10):1459–69. doi: 10.1007/s00439-021-02344-6 (PMC8460539; doi:10.1007/s00439-021-02344-6)
Supplement: Supplementary file 1 — Supplementary file1 (PDF 630 KB) [file 439_2021_2344_MOESM1_ESM.pdf]

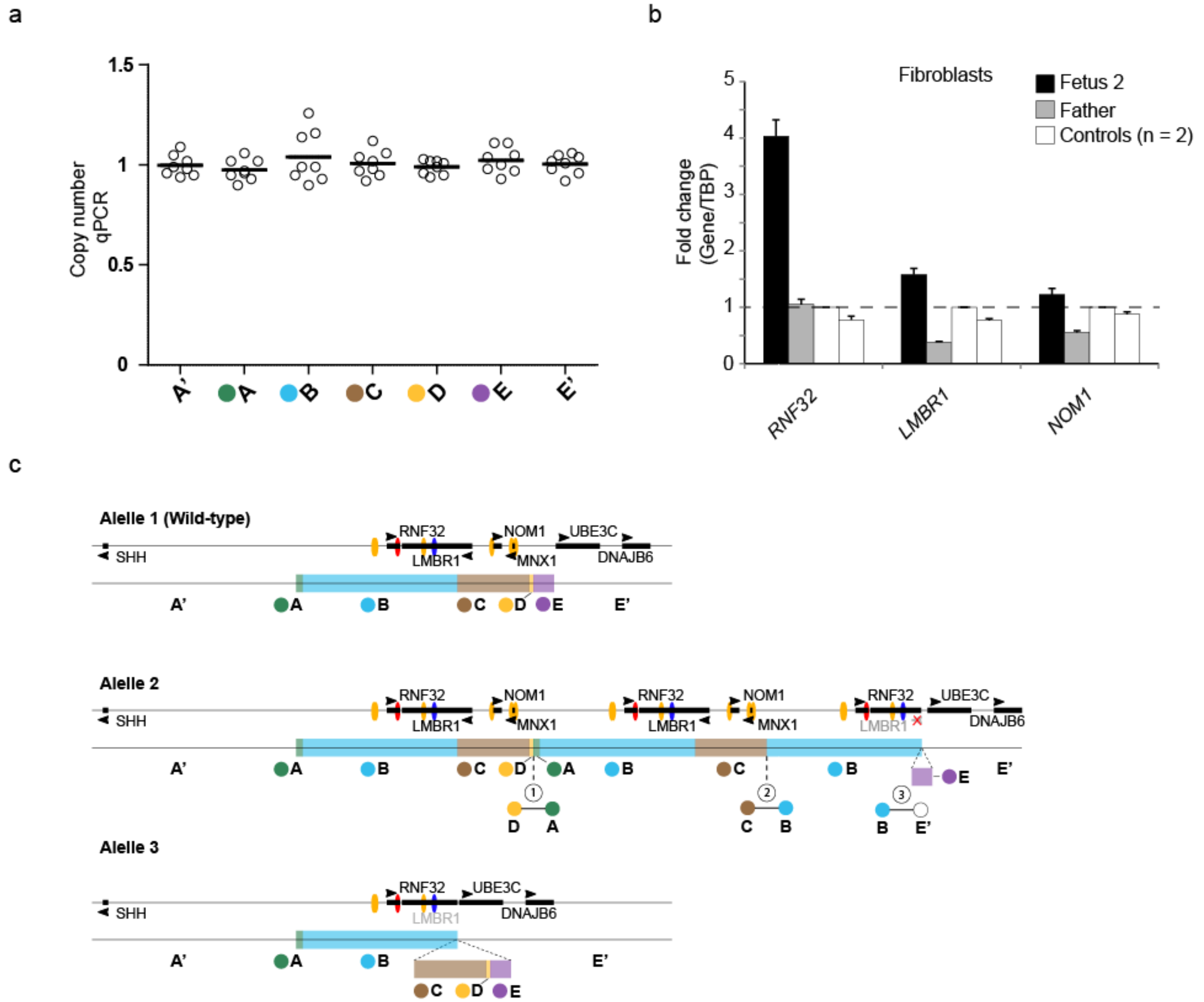

<u>Figure S1</u>: Quantitative PCR in samples from the studied family. (**a**) qPCR excluded the presence of the CGR in healthy relatives (paternal grandfather I-1, paternal grandmother I-2, mother II-2 and healthy female sibling III-2). Each dot represents the mean of three technical replicates per sample. Two qPCR replicates were performed per sample. The fragments A-E have different copy number values in the fetuses. Fragment A is shown in green, B in blue, C in brown, D in orange and E in purple. (**b**) RT-qPCR in fibroblasts from the Fetus 2 showed upregulation of *RNF32* and *LMBR1*, while *LMBR1* and *NOM1* were downregulated in the father, in comparison to two healthy controls. (**c**) Schematic representation of alleles likely to be present in the father's samples. The low copy number state of the C and D fragments in the father's samples is likely caused by the presence of a third allele, without the C, D and E fragments. In this scenario, the father is mosaic for three different combinations at different ratios (Allele 1 + Allele 2; Allele 1 + Allele 3; and Allele 2 + Allele 3). To achieve the copy number state observed in the father's

blood array CGH (**Fig. 2a**), 20% of his blood cells should consist of Allele 1 + Allele 2, 50% of Allele 1 + Allele 3 and 30% of Allele 2 + Allele 3. This mosaic scenario would explain the reduced *LMBR1* expression and the low copy number state of the C and D fragments in the father's samples.

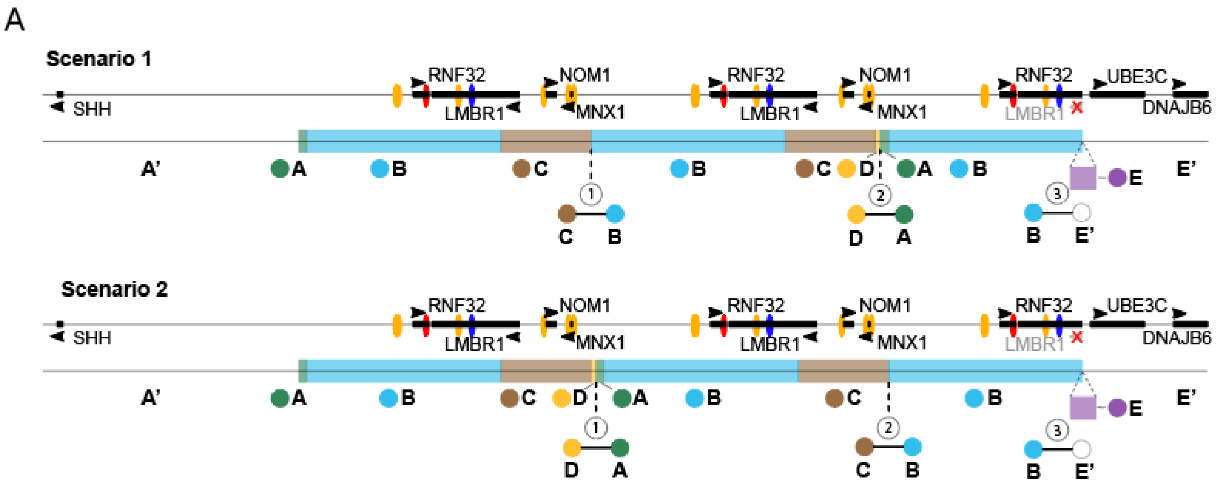

<u>Figure S2</u>: Genes and enhancers at the 7q36.3 region. (**a**) Genome sequencing breakpoint analysis disclosed two possible linear sequence scenarios. Scenario 1: A'-A-B-C-D-(1)-A-B-C-(2)-B-(3)-E' and Scenario 2: A'-A-B-C-(1)-B-C-D-(2)-A-B-(3)-E'.

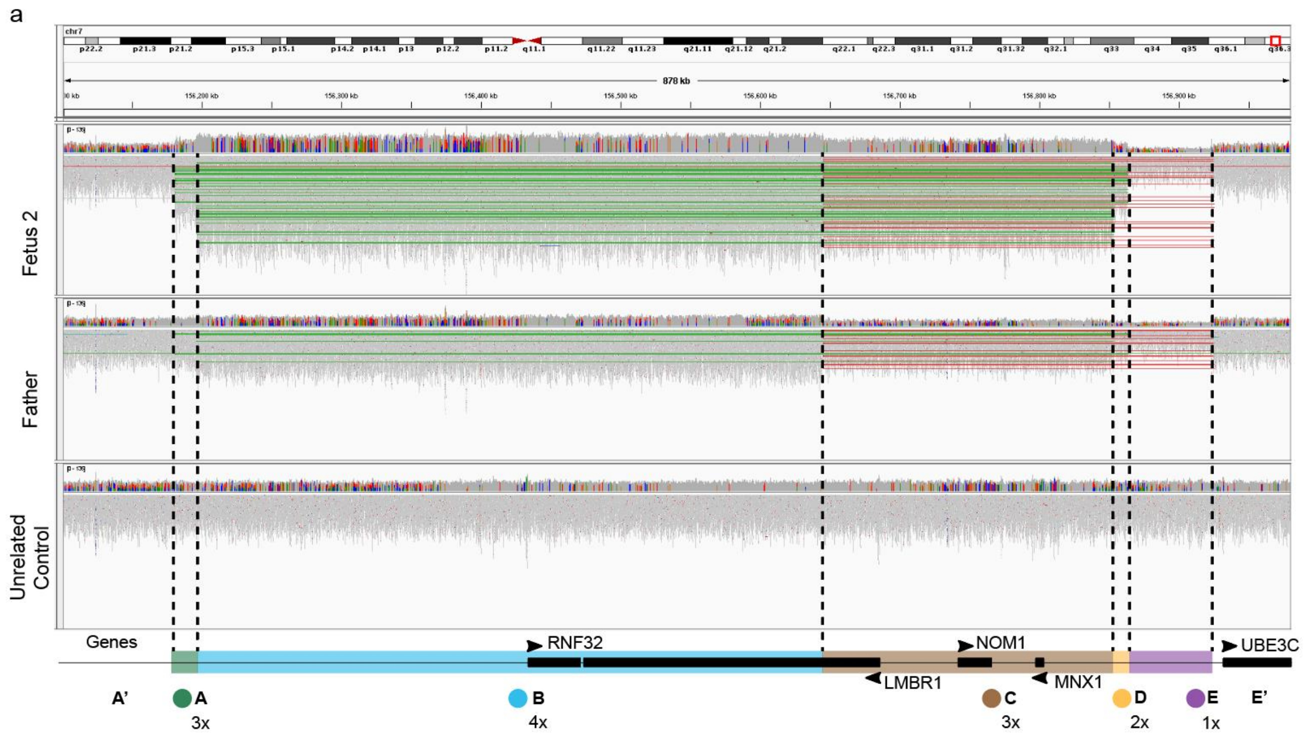

<u>Figure S3</u>: Genome sequencing results visualized using the Integrative Genomics Viewer (IGV) browser. (**a**) First and second panel: Split-reads in green show the duplication events in the Fetus 2 and the healthy father. Note that only few split-reads (green) are observed in the father GS, which supports mosaicism in his samples. Split-reads in red show the B-E' interaction. Last panel: GS from an unrelated healthy individual used as control.

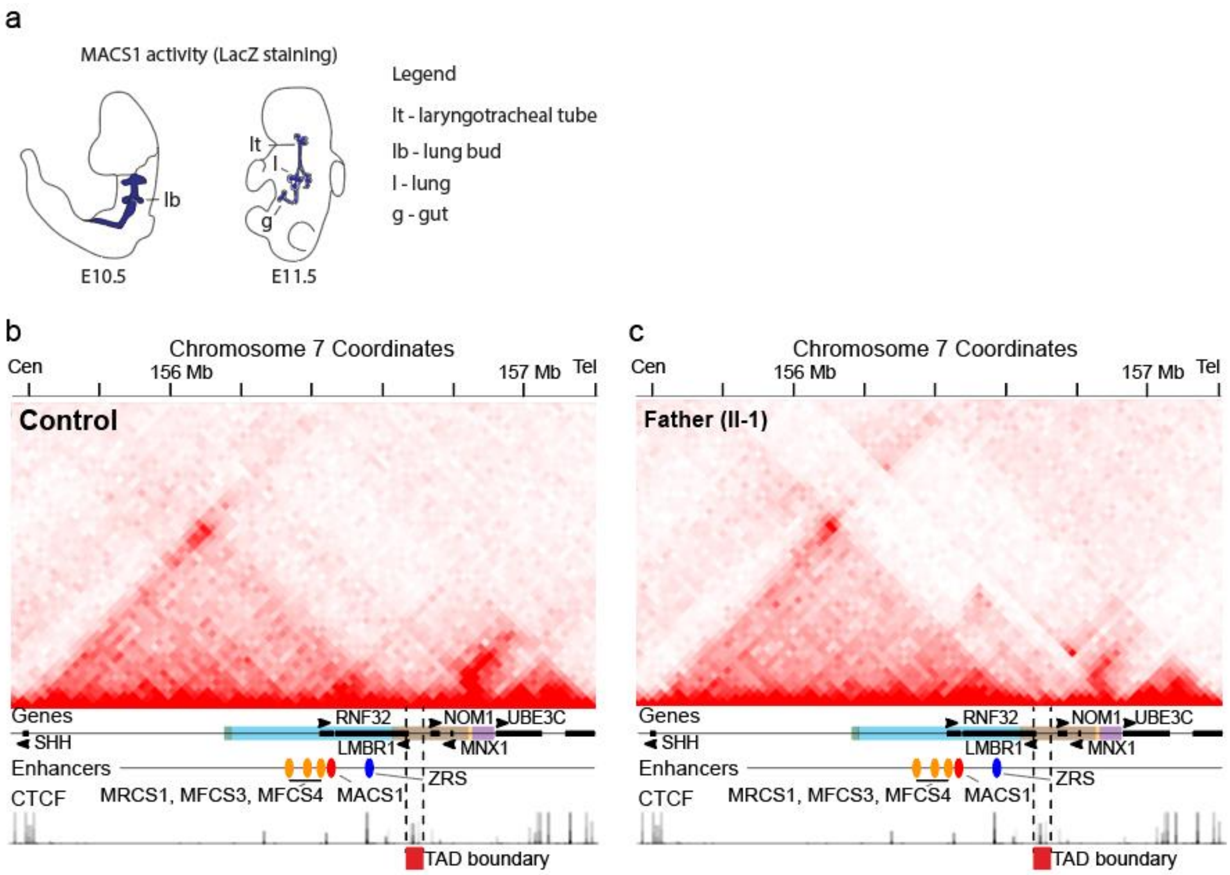

Figure S4: Enhancer activity and Hi-C maps. (A) Schematic representation of MACS1 enhancer activity, measured by LacZ staining, in mouse embryos at E10.5 and E11.5 (based on Sagai et al., 2009). (B) Control Hi-C map. The boundary separating both TADs (red square between the two dashed black lines) contains peaks of CTCF binding and it is located upstream the *LMBR1* transcription start site (TSS). ChIP-seq ENCODE data for CTCF (AG09309; human toe fibroblast from an apparently healthy adult). Note that the boundary is "located" in the C fragment. (B-D) The first neo-TAD is 680 kb long and the only way of creating this neo-TAD is by having C-D-A-B sequence in this order, followed by another C fragment, which contains the boundary. (C) Father Hi-C map showing a similar, but weaker, signal of ectopic chromatin interaction.
